# Perioperative nutrition practices in gastrointestinal cancer surgery: A nationwide survey among German surgical departments

**DOI:** 10.1007/s00423-025-03906-2

**Published:** 2025-11-10

**Authors:** Rahel Maria Strobel, Katharina Beyer, Johannes Christian Lauscher, Marc Martignoni, Christoph Reißfelder, Tim Vilz, Arved Weimann, Maria Wobith

**Affiliations:** 1 Department of General and Visceral Surgery, Charité – Universitätsmedizin Berlin, corporate member of Freie Universität Berlin und Humboldt-Universität zu Berlin, Campus Benjamin Franklin, Berlin, Germany; 2 Department for General, Visceral and Transplantation Surgery, University Medicine Augsburg, Stenglinstraße 2, 86156 Augsburg, Germany; 3 Department of Surgery, University Hospital rechts der Isar, Technical University of Munich (TUM), Ismaninger Str. 22, 81675 Munich, Germany; 4 Department of Surgery, Medical Faculty Mannheim, University Medical Center Mannheim, Heidelberg University, Theodor-Kutzer-Ufer 1-3, 68167 Mannheim, Germany; 5 Department of General, Visceral, Thoracic, and Vascular Surgery, University Hospital Bonn, Rheinische Friedrich-Wilhelms-University Bonn, Venusberg-Campus 1, 53127 Bonn, Germany; 6 Department of General, Visceral, and Oncological Surgery, St. George Hospital, Leipzig, Germany; 7 Department of Surgery, National University Hospital, Singapore, Singapore

**Keywords:** ERAS, Perioperative nutrition, Gastrointestinal surgery, Gastrointestinal cancer

## Abstract

**Background:**

Perioperative nutrition is a cornerstone of enhanced recovery in gastrointestinal cancer surgery, with international guidelines recommending early oral intake and standardized screening. This study aimed to assess current perioperative nutrition practices in German surgical departments and evaluate their alignment with guideline-based recommendations.

**Methods:**

A nationwide cross-sectional survey was conducted between September 18, 2024, and January 2, 2025, involving surgical departments that perform major gastrointestinal cancer resections. The 93-item anonymous questionnaire addressed pre- and postoperative nutrition strategies related to esophagectomy, gastrectomy, pancreatoduodenectomy and colorectal resections. Descriptive statistics were used to analyse the responses.

**Results:**

A total of 263 hospitals participated in the survey. More than one-third of hospitals (35.1%) reported no routine preoperative malnutrition screening and only 6.7% performed a structured nutritional assessment. There was no consistent agreement on postoperative feeding strategies including the timing of oral intake especially in upper gastrointestinal surgery. Nasogastric tubes were routinely placed postoperatively in 66 .1% of gastrectomies, 63.5% of esophagectomies, and 64.6% of pancreatoduodenectomies, but timing of postoperative removal varied widely. Hospitals with higher levels of care (e.g. university or maximum care hospitals) were significantly more likely to perform routine malnutrition screening (*p* = 0.002) and to allow early drinking after colorectal surgery (*p* < 0.001). The presence of structured nutrition support teams was associated with higher rates of guideline-compliant preoperative screening (76.3% vs. 47.4%; *p* < 0.001).

**Conclusion:**

Perioperative nutrition practices in German gastrointestinal cancer surgery vary considerably and often deviate from established guidelines.These findings underline the need for greater standardization and broader adoption of evidence-based perioperative nutrition strategies to ensure optimal patient outcomes.

**Supplementary Information:**

The online version contains supplementary material available at 10.1007/s00423-025-03906-2.

## Introduction

Malnutrition is common among patients undergoing major gastrointestinal (GI) surgery and is strongly associated with adverse outcomes [1]. In cancer-related gastrointestinal resections, up to two-thirds of patients may be malnourished preoperatively [2]​, which significantly increases the risk of postoperative complications, lengthens hospital stay, and worsens survival. Optimizing nutritional status in the perioperative period can improve surgical recovery​, making perioperative nutrition a cornerstone of modern surgical care [1]. Enhanced Recovery After Surgery (ERAS) protocols have accordingly integrated nutritional elements as part of a multimodal strategy to reduce surgical stress and improve outcomes​ [1].

International and national guidelines such as ERAS^®^Society, the European Society for Clinical Nutrition and Metabolism (ESPEN), and the German AWMF guidelines uniformly advocate for a standardized approach to perioperative nutrition [1, 3–7]. Key recommendations include routine screening of all surgical patients for malnutrition risk, comprehensive nutritional assessment for those at risk, and timely nutritional interventions (e.g. oral or enteral feeding initiated within 24 h after surgery). ERAS guidelines stress the importance of avoiding prolonged fasting and commencing oral intake as early as possible postoperatively [5–7].

However, despite these evidence-based guidelines, perioperative nutritional management in routine practice often remains heterogeneous. Anecdotal reports and earlier studies suggest substantial variability in how hospitals implement nutritional care, with practices frequently differing between institutions or even between individual surgeons [8].

In Germany, the extent to which evidence-based nutritional recommendations have been implemented in surgical practice has not been investigated in recent years. Prior surveys over a decade ago found variability in the adoption of ERAS elements in colorectal surgery​ [8], but there is a paucity of contemporary data focusing specifically on nutrition strategies. Meanwhile, healthcare trends such as centralization of complex cancer surgery (with volume-based certification of centers) could influence nutritional practices and outcomes. To address these knowledge gaps, we conducted a nationwide cross-sectional survey of surgical departments performing major GI cancer resections (esophagectomy, gastrectomy, pancreatoduodenectomy, and colorectal resection). Our primary goal was to characterize current perioperative nutritional strategies and identify areas of consensus versus divergence in practice, covering domains including preoperative malnutrition screening, formal nutritional assessment, calculation of caloric/protein requirements, postoperative feeding protocols, and the use of adjunctive support (feeding tubes or parenteral nutrition). By comparing real-world practices against guideline recommendations, the survey provides insight into how consistently evidence-based nutrition care is applied in German surgical units and highlights critical areas where lack of consensus may signal the need for standardization, quality improvement initiatives, or further research.

## Methods

We conducted a cross-sectional survey of surgical departments performing major gastrointestinal cancer resections in Germany. The following surgical procedures were considered: esophagectomy, subtotal and total gastrectomy, pancreatoduodenectomy and pancreatectomy as well as colorectal resection including right- or left-sided hemicolectomy, colectomy and rectal resection with or without ileostomy.

The survey questions were designed to address pre- and postoperative management related to nutritional screening, assessment, and intervention. The questionnaire, consisting of 93 items, was validated by five surgeons (M. W., R. S., J. L., K. B., A. W.) from the CA PeriVis of the DGAV (Surgical Working Group for Perioperative Management in Visceral Surgery of the German Society of Surgery) and by K. E. – a dietitian at the Department of Gastroenterology of Charité – Universitätsmedizin Berlin, Campus Benjamin Franklin. She provides pre- and postoperative nutritional counselling for patients with gastrointestinal cancer. A stepwise approach was used in which procedure-specific questions were asked only if the corresponding surgery was performed at the hospital of the answering person.

The nationwide survey was conducted between September 18, 2024, and January 2, 2025. It was anonymous.

The survey can be divided into the following six thematic blocks:

1. General information about the answering person and performing surgeries.
2. Screening for malnutrition and nutritional assessment (nutritional team, nutritional counselling, caloric and protein intake).
3. Nutritional management in colorectal cancer surgery.
4. Nutritional management in gastric cancer surgery.
5. Nutritional management in esophageal cancer surgery.
6. Nutritional management in pancreatic cancer surgery.

In the sections addressing various organ-specific surgical procedures, the first question asked whether patients were permitted to eat and drink freely on the day of surgery. If answered affirmatively, further details were collected regarding what patients were allowed to consume and from what time on the day of surgery. Additionally, respondents were asked what patients were allowed to eat on the first postoperative day. For all organ groups, the timing of the initiation of parenteral nutrition was also assessed. Multiple predefined response options, including “individualized,” were provided.

In the colorectal surgery section, participants were asked whether dietary advancement differed in cases where an ileostomy was created. For the gastric, esophagus, and pancreas groups, information was collected on the frequency of meals offered and the use of a nasogastric tube postoperatively. If a nasogastric tube was used, respondents were further asked when enteral nutrition was initiated and when the tube was removed. In the esophageal surgery section, additional questions included whether an intraoperative needle catheter jejunostomy was placed and when it was subsequently removed.

In addition, compliance with the recommendations of various guidelines was assessed. These included ERAS (enhanced recovery after surgery) guideline for colorectal resection [7], ERAS guideline for gastric cancer [3], ERAS guideline for oesophageal resection [5], ERAS guideline for pancreatoduodenectomy [6], ESPEN guideline (European society for clinical nutrition and metabolism) [1, 4], guideline on Clinical Nutrition in Surgery of the German Society for Nutritional Medicine (DGEM) [9], German AWMF S3 guideline for gastric [10]and oesophageal cancer [11]and POMGAT guideline (perioperative care for gastrointestinal tumours) [12]. Agreement between hospitals was considered to be present when 75% of them adhered to the respective guideline recommendation.

The answering hospitals were categorized into different levels of care. Classification was based on the number of beds as follows: minimum care hospital (150 to 199 beds), primary care hospital (200 to 299 beds), standard care hospital (300 to 499 beds), central care hospital (500 to 699 beds), maximum care hospital (700 to over 1000 beds) and university hospital.

Questions related to specific surgical procedures appeared in the survey only if it was indicated that such procedures were performed in the respondent`s hospital. The questionnaire was analyzed even if not all 93 questions were answered. Thus, the absolute number of answers per question could vary. In cases where two or more participants from the same hospital completed the survey, only one response was analyzed. As respondents were asked to indicate their position within the hospital, the response from the person holding the highest position was selected for evaluation.

The online survey was sent via the mailing list of the “German Society of General and Visceral Surgery” (DGAV) which comprises 4,800 members. Furthermore, the network of the German association “Die Chirurginnen” (female surgeons) was used. Approximately 700 individuals were reached through this process. Furthermore, we directly addressed hospitals with a certificate of the “German Cancer Society” and cooperating hospitals of our own clinics, addressing approximately another 400 persons. Among those individuals, there may be some degree of overlap. This may have resulted in some individuals being contacted twice via email.

Study data were collected and managed using REDCap electronic data capture tools hosted at Charité – University Berlin, Campus Benjamin Franklin [13, 14]. REDCap (Research Electronic Data Capture) is a secure, web-based software platform designed to support data capture for research studies, providing an intuitive interface for validated data capture, audit trails for tracking data manipulation and export procedures, automated export procedures for seamless data downloads to common statistical packages and procedures for data integration and interoperability with external sources.

Statistical analysis was performed using IBM SPSS Statistics 30^®^ (IBM, Armonk, New York, USA). Outcome variables were the items of the survey. Chi-square test was performed to compare guideline recommendation with actual practice. Parameters were depicted according to their scale and distribution with absolute and relative frequencies. *P* values ≤ 0.05 were considered as statistically significant when considering the compliance with current guidelines.

## Results

In total, 306 responses were received. After excluding 43 duplicate submissions originating from the same hospitals, the final analytic sample comprised 263 unique hospitals. The median age was 47 years (25% IQR = 38 years; 75% IQR = 55 years). More than two-thirds of participants worked in leadership positions in the hospital (71.8%) and the most common specialization among participants was visceral surgery (*n* = 200, 76%) (see supplement Table 1).

### Number of elective cancer surgeries

A total of 257 (97.7%) hospitals performed elective colorectal cancer surgery, 216 (82.1%) gastric cancer surgery, 155 (58.9%) pancreatic cancer surgery and 77 (29.3%) oncologic esophageal resections (see supplement Fig. 1). Of those hospitals performing pancreatic cancer surgery, 60% had a certificate of “Deutsche Krebsgesellschaft” (German Cancer Society). In colorectal cancer surgery, a certificate was available in 58.8%. Only 37.7% were certified for esophageal cancer and 18.1% for gastric cancer by the German Cancer Society (see supplement Fig. 1). Case numbers per hospitals are displayed in Fig. 2 in the supplement.

### Nutritional management

A total of 194 participants (73.8%) responded to the questions regarding nutritional standards in their clinical practice. In 118 hospitals (60.8%) included in the survey, a dedicated nutrition support team (NST) is available, and about half of these teams meet the requirements for conducting “nutritional medicine complex treatment,” coded as OPS 8-98j in Germany. In our survey, participating clinics reported that postoperative diet progression was most commonly based on internal hospital standard operating procedures (74.5%). In 45.2% of cases, this decision was made individually by the surgeon, and in 9.9% by the nutrition support team. Additionally, 39.9% indicated that progression was guided by the patient’s tolerance, while decisions by the nursing team (1.1%) or based on patient age (2.3%) were rare. Nineteen of the 263 participating hospitals (7.2%) held an ERAS certification and took part in the study, selected from the 35 ERAS-certified hospitals currently accredited in Germany.

**Fig. 1:**
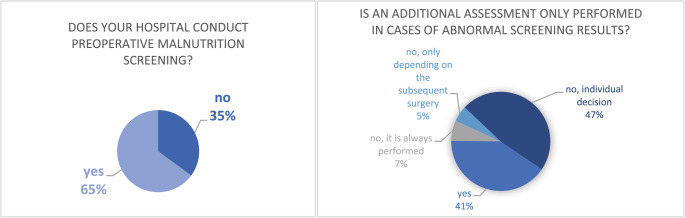
Proportions of hospitals performing screening for malnutrition and additional assessment

Malnutrition screening is performed in 126 hospitals (64.9%), as illustrated in Fig. 1, with the majority utilizing NRS-2002 as the primary screening tool (*n* = 113, 89.7%). Other screening tools include MUST (*n* = 8, 6.3%), MNA short-form (*n* = 6, 4.8%), SGA (*n* = 4, 3.2%), and PG-SGA (*n* = 3, 2.4%). Additionally, four hospitals (3.2%) reported using alternative screening tools, including self-created questionnaires, total serum protein levels, and different approaches based on the planned surgical procedure. In cases where malnutrition screening results are positive, 79 hospitals (40.7%) conduct an additional nutritional assessment. In 92 hospitals (47.4%), the need for assessment is determined on an individual basis (see Fig. 1). Tools used for nutritional assessment are displayed in Table 2 in the supplement.

A total of 76 hospitals (39.2%) calculate calorie requirements using nutritional calculators (*n* = 44, 58.7%), reference values (*n* = 41, 54.7%), bioelectrical impedance analysis (BIA) (*n* = 7, 9.3%), indirect calorimetry (*n* = 5, 6.7%), or direct calorimetry (*n* = 4, 5.3%), performed at varying time points between hospitals, see Table 2 in the supplement. Regarding dietary options, 179 hospitals (92.3%) offer at least five different types of diets for their patients. In 61 hospitals (31.4%), there is no postoperative monitoring of actual calorie consumption. Caloric intake is estimated in 92 hospitals (47.4%), while in 59 hospitals (30.4%), patients are asked about their actual food intake, and 19 hospitals (9.8%) use plate diagrams for assessment.

Dietary counselling is implemented in most hospitals on a routine basis. Notably it is more often performed in upper GI surgery than in colorectal surgery: for colorectal resections (*n* = 113, 52.3%), gastric resections (*n* = 139, 82.7%), pancreatic resections (*n* = 95, 84.1%), and oesophageal resections (*n* = 47, 90.4%). The primary provider of nutritional counselling across all types of surgical procedures is a dietitian (78.9–88.5%), followed by the attending physician (29.6–35.5%) and the nutritionist (25.6–36.5%). In some hospitals, a physician with a specialization in nutritional medicine is responsible for counselling (12.6–21.2%), while in others, the nursing team takes on this role (7.7–20.8%).

### Postoperative oral feeding

The postoperative nutritional regimen was assessed based on the types of surgeries performed in each hospital. The percentages presented refer specifically to hospitals that carry out these procedures.

Most hospitals performing colorectal resections allow patients to drink freely immediately after surgery (*n* = 201, 92.6%). However, free fluid intake postoperatively is less common in pancreatic surgery (*n* = 82, 72.6%), gastric surgery (*n* = 85, 50.0%), and oesophageal surgery (*n* = 15, 28.8%). In pancreatic surgery, 13 hospitals (11.5%) allow free drinking only if individually approved, a practice also followed in 25.3% of hospitals performing gastric resections (*n* = 43) and in 25.0% of hospitals conducting oesophageal resections (*n* = 13).

**Fig. 2:**
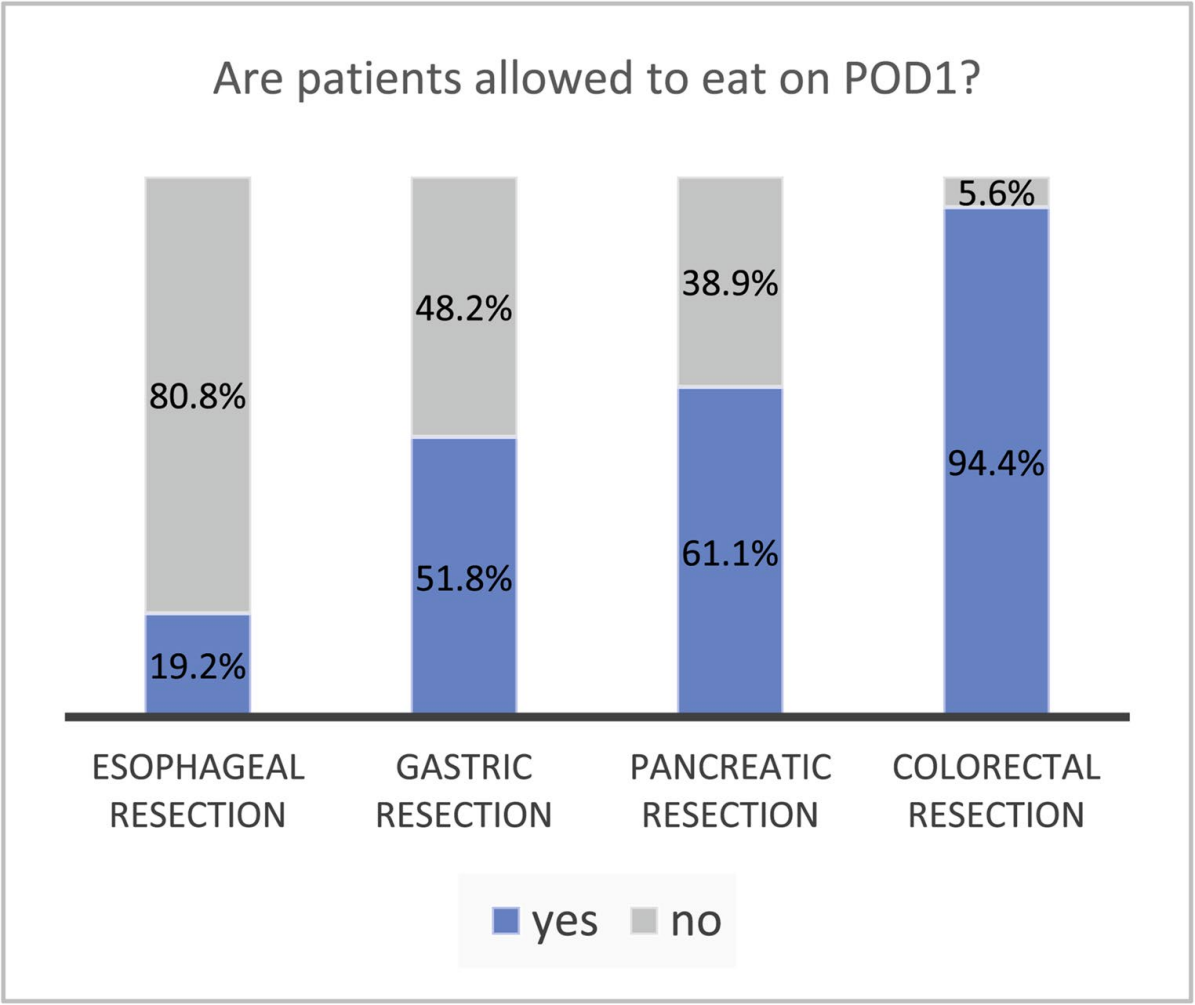
Proportion of hospitals that offer oral feeding (any kind of food including liquid and soft diet) on postoperative day (POD) 1

In 94.4% of hospitals performing colorectal resections (*n* = 203), patients are allowed to eat immediately and within 24 h after surgery. The timing of first oral intake in colorectal resection patients varies, with most hospitals allowing food after 4 h (24.3%), 6 h (26.2%), or on an individual basis depending on nausea (28.8%). Oral feeding within 24 h after surgery is practiced in 61.1% of hospitals performing pancreatic resections (*n* = 69), 51.8% of hospitals performing gastric resections (*n* = 87), and 19.2% of hospitals performing oesophageal resections (*n* = 10), as shown in Fig. 2. Table 1 provides an overview on what hospitals offer to eat and drink on POD 1. When patients were not allowed to eat on postoperative day 1 an individualized approach according to surgeons’ discretion or depending on the patient’s clinical condition was performed (see Table 3 in supplement).

**Table 1:** Choice of oral intake on postoperative day 1 (POD 1)

Choice of oral intake	Colorectal resection	Gastric resection	Oesophageal resection	Pancreatic resection
Tea	92.6%	93%	89.3%	94.7%
Coffee	52.1%	18.8%	14.3%	22.1%
High-calorie nutritional drink	76.6%	66.7%	-	81.3%
Broth	84.1%	75%	-	93.8%
Plain yoghurt	87.9%	58.3%	-	87.5%
Fruit yoghurt	62.6%	33.3%	-	75%
Milk soup	48.6%	58.3%	-	75%
Fruit or vegetable puree	38.3%	25%	-	56.3%
Toast or white bread	18.7%	8.3%	-	56.3%
Pureed food	13.1%	8.3%	-	25%
Light full diet	7.5%	0%	-	0%

Further oral feeding is introduced on POD 2 and 3 (66.7% in colorectal resections, 31.8% in pancreatic resections, 54.9% in gastric resections, 14.3% oesophageal resections), or after POD 3 (22.7% in pancreatic resections, 25.6% in gastric resections, 23.8% oesophageal resections). Postoperative oral feeding also depends on factors such as checking for anastomosis via endoscopy or contrast swallow in 50.0% of hospitals performing oesophageal resections (*n* = 21) and in 32.9% in hospitals doing gastric resections (*n* = 27).

For gastric resections, 57 hospitals (33.5%) have different postoperative oral feeding protocols for patients undergoing subtotal versus total gastrectomy. This variation also applies to colorectal resections, where diet progression differs when a stoma has been created (*n* = 117, 54.2%). In 15 hospitals (6.9%), patients with a stoma are allowed to eat immediately after surgery, unlike those without a stoma. Additionally, 7 hospitals (3.2%) offer a specialized diet for stoma patients.

Following pancreatic resection, dietary standards vary among hospitals in cases of pancreatic fistula. In most hospitals (*n* = 70, 61.9%), diet progression is individualized based on the patient’s tolerance. However, in 51 hospitals (45.1%), an oral diet is allowed. A more restrictive approach is followed in 15 hospitals (13.3%), where only clear liquids are permitted. In 12 hospitals (10.6%), a diet rich in medium-chain fatty acids (MCT) is provided, while 9 hospitals (8.0%) rely solely on parenteral nutrition. Additionally, one hospital each (0.9%) follows a nil-per-mouth protocol, allows only unsweetened liquids, or administers enteral nutrition exclusively via a feeding tube.

### Nasogastric/-jejunal tube

**Fig. 3:**
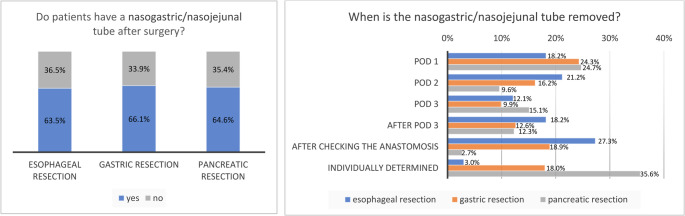
Proportion of hospitals that routinely use nasogastric/nasojejunal tubes and the timing of their removal

Around two-third of patients receive a nasogastric/nasojejunal tube after surgery of the stomach, oesophagus or pancreas. The nasogastric tube is placed solely for decompression in 42.4% (*n* = 14) of hospitals performing oesophageal resections. The removal of nasogastric/nasojejunal tubes varies depending on the procedure and hospital protocol, displayed in Fig. 3.

### Enteral tube feeding

Enteral feeding via nasogastric/nasojejunal tube varies between hospitals and surgery as displayed in Table 2. In Gastric and pancreatic surgery over 40% start with enteral tube feeding on the first postoperative day.

**Table 2:** Timing of enteral tube feeding

Gastric surgery	*N* = 111
On the day of surgery	9 (8.1%)
On POD 1	45 (40.5%)
In case of complications	9 (8.1%)
No enteral feeding via nasojejunal tube	38 (34.2%)
Not at all, as oral diet is taking place despite nasojejunal tube	10 (9.0%)
Oesophageal surgery	*N* = 33
On the day of surgery	2 (6.1%)
On POD 1	4 (12.1%)
After POD 1	4 (12.1%)
In case of complications	5 (15.2%)
No enteral feeding via nasogastric tube (solely for decompression)	14 (42.4%)
Enteral feeding is provided via feeding jejunostomy	4 (12.1%)
Pancreatic surgery	*N* = 73
On the day of surgery	8 (11.0%)
On POD 1	33 (45.2%)
In case of complications	6 (8.2%)
No enteral feeding via nasogastric tube	22 (30.1%)
Not at all, as oral diet is taking place despite nasogastric tube	4 (5.5%)

A needle catheter jejunostomy is routinely placed in 4.8% of hospitals (*n* = 8) after gastric resection, in 15.4% of hospitals (*n* = 8) after oesophageal resection, and in 3.5% (*n* = 4) after pancreatic resection. In some hospitals, jejunostomy placement depends on intraoperative surgical findings (gastric resection: 11.3%, *n* = 19; oesophageal resection: 9.6%, *n* = 5; pancreatic resection: 4.4%, *n* = 5), the patient’s nutritional status (gastric resection: 14.3%, *n* = 24; oesophageal resection: 9.6%, *n* = 5; pancreatic resection: 1.8%, *n* = 2), or the patient’s overall risk profile (gastric resection: 11.3%, *n* = 19; oesophageal resection: 11.5%, *n* = 6; pancreatic resection: 7.1%, *n* = 8).

If a needle catheter jejunostomy is placed intraoperatively, the removal takes place after discharge in most cases (gastric resection: 42.6%, *n* = 20; oesophageal resection: 62.5%, *n* = 15; pancreatic resection: 52.6%, *n* = 10).

### Parenteral nutrition

There is a wide variation in standards for administering parenteral nutrition across hospitals in Germany. The most commonly followed standard is providing parenteral nutrition when “complications arise, and oral feeding is not possible”. This is followed by three other frequently selected criteria: “in cases of preoperative malnutrition,” “if patients are unable to consume more than 50% of their caloric needs orally within seven days postoperatively,” and “as a supplementary measure until oral or enteral feeding fully meets caloric requirements”. The details are presented in Fig. 3 in the supplement.

### Are there differences between the hospitals regarding the compliance with current recommendations?

Overall, 217 hospitals answered the questions about drink and food intake after colorectal surgery. There was a significant difference in the responses based on the hospital’s level of care regarding drinking after colorectal resection, with higher-level care hospitals allowing patients to drink sooner (*p* < 0.001). There was no correlation between the level of care of hospitals and the permission to eat immediately after colorectal resection (*p* = 0.769), but a trend that eating directly after colorectal resection is more likely to be allowed in hospitals with case numbers > 100 colorectal resections per year (*p* = 0.056).

**Fig. 4:**
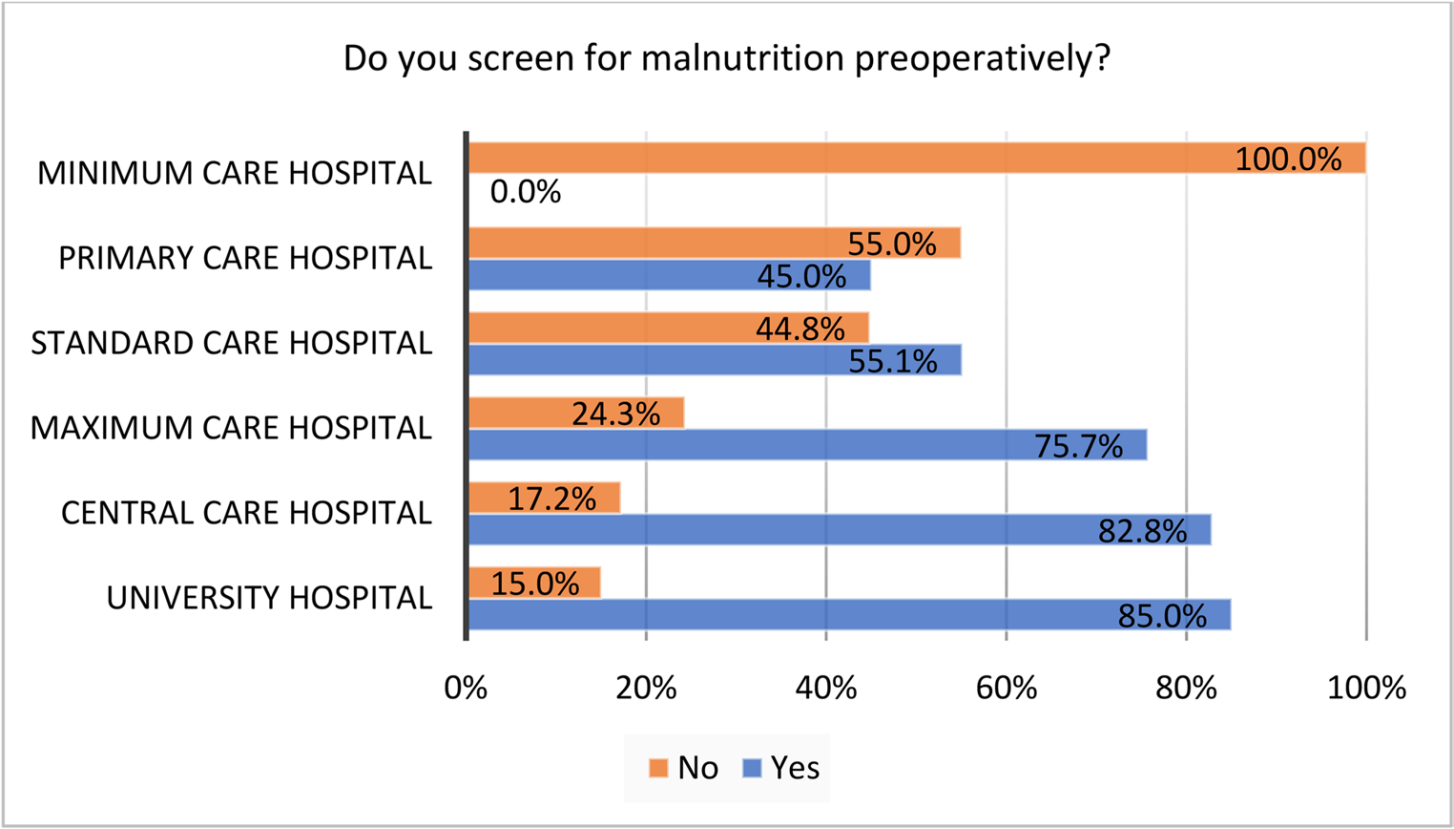
Screening of malnutrition based on the hospitals’ level of care

University hospitals, central care hospitals and maximum care hospitals screened more often for malnutrition preoperatively than hospitals with a lower level of care; *p* = 0.002 (see Fig. 4). If a nutritional support team existed in the hospital, preoperative screening for malnutrition was performed more often (76.3%) than without nutritional team (47.4%); *p* < 0.001.

#### Compliance with recommendations

**Table 3.**
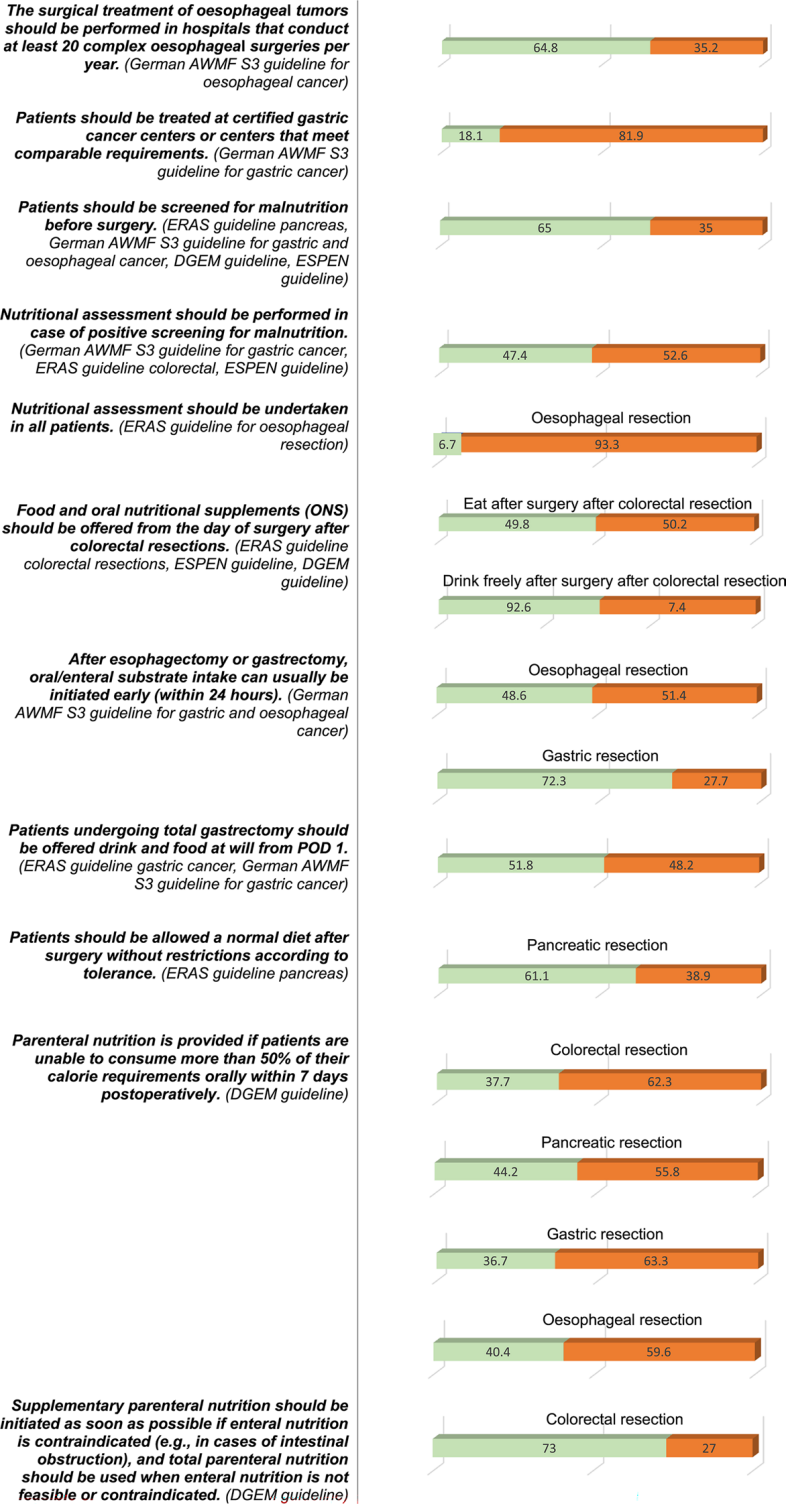

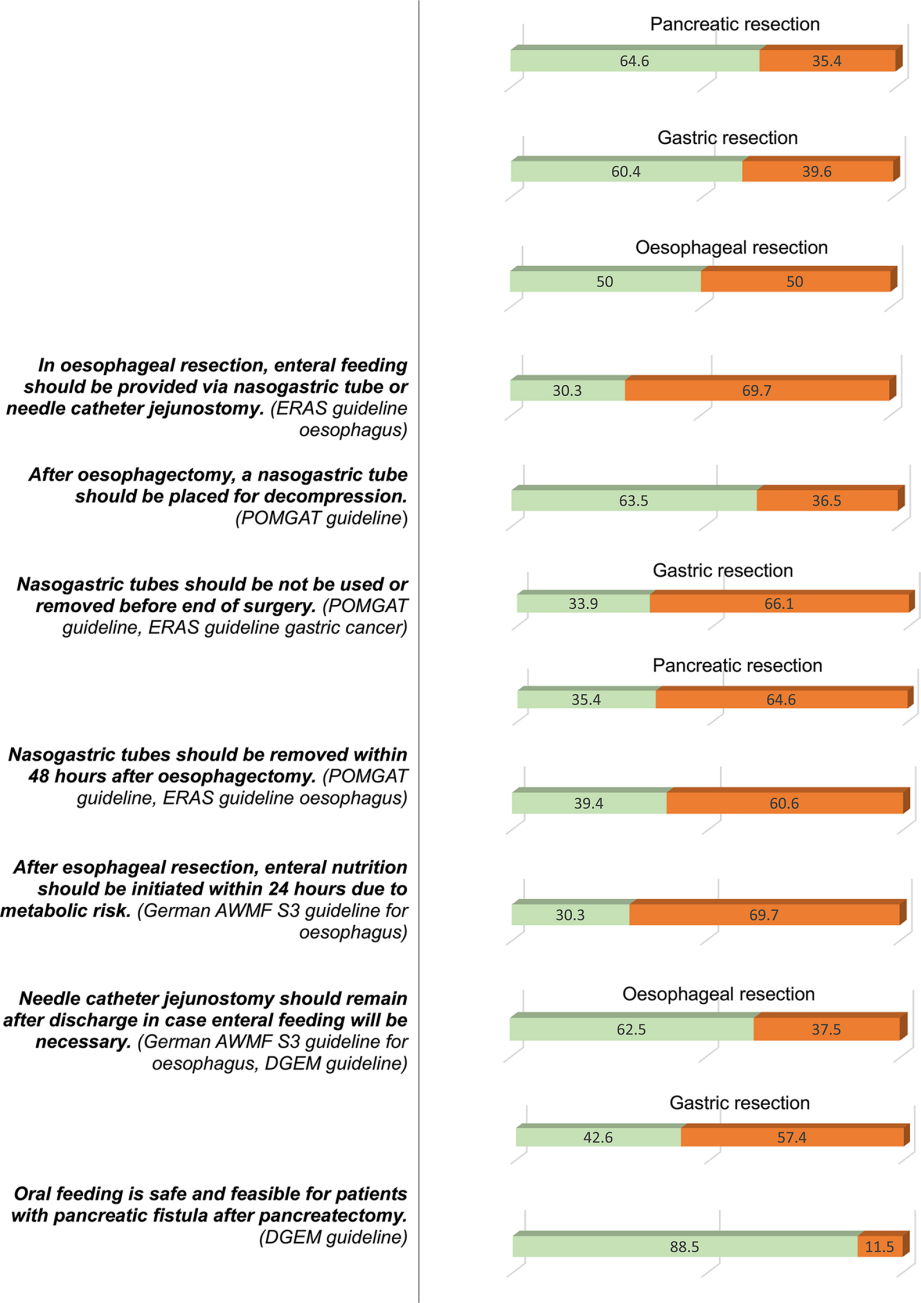
Provides an overview of compliance with the guideline recommendations addressed in the survey. Only two out of 28 statements (7.1%) reached consensus, with at least 75% of hospitals followed these recommendations: ‘Oral feeding is safe and feasible for patients with pancreatic fistula after pancreatectomy’ (DGEM), and ‘Oral intake of fluids should be offered on the day of surgery following colorectal resections’ (ERAS guideline for colorectal surgery, ESPEN, DGEM). Eleven statements (37.9%) were followed by at least half of the hospitals, while 16 recommendations (55.2%) were not implemented by the majority of hospitals. Agreement between hospitals was considered to be present when 75% of them adhered to the respective guideline recommendation

## Discussion

This survey provides an overview of current practices in perioperative nutrition management across German hospitals. Overall, the results reveal substantial variability in perioperative nutritional management, indicating that despite the presence of guidelines, a uniform approach is often lacking. Key aspects of care such as malnutrition screening, nutritional assessment, determination of nutritional needs, timing of postoperative feeding, and use of enteral or parenteral support differ markedly between institutions. This variability mirrors observations in other countries: a recent Korean survey found significant inconsistencies in perioperative nutrition practices, with many surgeons adhering to traditional habits despite ERAS recommendations [15] as well as a survey revealed lack in ability to manage malnutrition preoperatively in the UK [2].

### Variable implementation of nutritional screening and assessment

Malnutrition screening is performed in only 65% of the surveyed hospitals, despite strong recommendations from all major guidelines and societies [1, 16]. The most commonly used screening tool is the NRS-2002, which is well-validated, easy to implement and particularly suitable for surgical patients [17]. However, only 40.7% of hospitals proceeded with a comprehensive nutritional assessment when screening indicated risk, and just 6.7% had a policy of routinely performing detailed assessments in all patients (even those who were screened negative). Nearly half of the hospitals lack standardization and make assessment decisions on a case-by-case basis. The screening should trigger further evaluation and intervention for at-risk patients [18]. The low rate of comprehensive assessments implies that many patients identified as nutritionally “at risk” may not receive the full work-up needed to diagnose malnutrition or quantify its severity. Consequently, opportunities for nutritional optimization like prehabilitation could be missed.

This gap between guidelines and practice is corroborated by experiences in a 2021 UK survey of perioperative clinicians who found that while around 58% reported using a screening tool, only about half of them would then refer at-risk patients to a dietitian or nutritional support team​ [2]. These international parallels suggest that the challenge is not merely awareness of guidelines, but also the practical aspects of implementation.

### Postoperative feeding practices

One of the most striking areas of heterogeneity revealed by the survey is the timing and progression of postoperative oral feeding, particularly after major GI resections. ERAS guidelines consistently recommend initiating oral intake within 24 h postoperatively for most elective GI procedures, unless contraindicated [3, 6, 7]. Numerous studies across colorectal and upper GI surgery support early feeding, showing associations with faster bowel recovery and shorter hospital stays without increased anastomotic complications​ [19–21].

After colorectal resections, most hospitals allow unrestricted clear fluids on the day of surgery, reflecting widespread acceptance of this practice. However, only about half permit solid food on the same day, while the rest prefer to wait until POD1. By POD 1, nearly all centers begin solids, indicating general consensus to initiate full oral feeding within 24 h post-op.

In contrast, postoperative nutrition practices following upper GI surgeries (gastrectomy, pancreatoduodenectomy, esophagectomy) vary significantly. Our survey showed no clear consensus: about half the hospitals allow oral intake (at least sips or liquids) on POD 1 after gastrectomy or pancreatic surgery, while the rest delay further. This is at odds with evidence and guidelines recommending early feeding. For gastrectomy, multiple RCTs and meta-analyses have demonstrated that early oral intake (within 24 h) improves recovery—reducing time to first flatus and hospital stay—without increasing complications [19]. Notably, nearly 75% of surveyed centers begin some form of enteral feeding (oral or via tube) within 24 h, consistent with the German AWMF S3 guideline for gastric cancer, which calls for early enteral nutrition. The key difference lies in the route—oral versus tube—closely linked to practices surrounding nasogastric or jejunal tubes.

Practice variation is even more pronounced for esophagectomy. There is no clear agreement on when to start oral intake, with responses ranging from POD 1 to after POD 3. About half of the hospitals restrict oral feeding until after confirming anastomotic integrity—usually through radiologic contrast swallow or endoscopy. The rest are split between early feeders (POD 1–2) and those initiating around POD 3. While major guidelines advocate early enteral nutrition post-esophagectomy, they encourage tube feeding (via jejunostomy or nasogastric tube) if oral intake is delayed [5].

Although no guideline mandates routine anastomosis checks before oral feeding, many centers continue this practice to detect potential leaks early. However, growing evidence supports the feasibility of early oral intake post-esophagectomy without increasing leak risk [22]. One randomized trial comparing early vs. delayed oral feeding (with both groups receiving equivalent nutrition via oral diet or jejunostomy) found no difference in major complications​ [23]. Early oral intake was non-inferior in safety and avoided the discomfort of nil-by-mouth and prolonged tube feeding. However, a trade-off was noted: patients fed orally lost more weight—by 4 weeks post-op, the early-oral group lost an average of 8.0% body weight vs. 5.1% in the tube-fed group [23]. Reflecting this tension, about one-third of hospitals in our survey routinely use a feeding jejunostomy and delay oral intake, likely to better support nutritional status.

Monitoring of postoperative intake is often lacking. About one-third of hospitals do not systematically document oral intake, while many rely on subjective measures like visual estimates or patient reports. Only a minority use objective tracking tools (e.g. caloric data from hospital kitchens or intake-output charts). This is concerning, as patients often consume less than 30% of their caloric needs during the first postoperative week, particularly after upper GI surgery [23]. Failure to meet ESPEN targets (≥ 25 kcal/kg/day and ≥ 1 g protein/kg/day) has been linked to deterioration in nutritional status during cancer treatment [24]. Guidelines recommend close intake monitoring in the first postoperative week and escalation of nutritional support if intake falls below 50% of requirements [1, 4].

### Use of feeding tubes and parenteral nutrition

Our survey results underscore that the use of nasogastric (NG) or nasojejunal (NJ) tubes remains commonplace in many German surgical units, despite ERAS recommendations to minimize their routine use. Approximately two-thirds of hospitals performing esophagectomies, gastrectomies, or pancreatoduodenectomies reported routine placement of an NG/NJ tube. The timing of NG/NJ removal varied widely, indicating no consensus – some surgeons remove it early (within 1–2 days), others leave it longer. This approach differs notably from the principles of fast-track care [3, 6].

Beyond decompression, NG/NJ tubes in some cases were used as a feeding route. For esophagectomy patients, about 33% of hospitals use NJ tube feeding routinely, while nearly half of the patients received a feeding jejunostomy, depending on their individual risk profile. Unlike pancreatoduodenectomy, where routine NG tube use is not recommended routinely, the ERAS guidelines do recommend NJ tube placement after esophagectomy for decompression [5, 6]. For pancreatoduodenectomy patients, about half of hospitals provide enteral feeding via NG tube postoperatively.

When it comes to parenteral nutrition (PN), our survey reveals an expected lack of consensus except in clear-cut scenarios. Virtually all respondents agreed that if a patient develops complications that render the enteral route non-functional, PN should be initiated, which aligns with guidelines [1, 4]. Outside of such scenarios, practices diverged. Some hospitals favor early supplemental PN (for example, starting PN by postoperative day 3–4 if oral intake is inadequate), whereas others avoid PN unless absolutely required. This dichotomy is seen in other surveys as well. In Korea, for instance, 49% of surgeons reported routinely prescribing PN after major GI surgery​, even though international guidelines caution against routine use of PN when enteral feeding is feasible​ [15]. A prospective Australian study indicated that initiating nutritional support—either enteral or parenteral—on the first postoperative day in patients who were required to remain nil by mouth is associated with a reduction in surgical complications [25].

### Role of institutional factors

The survey allowed us to explore whether certain institutional characteristics correlated with better guideline adherence in perioperative nutrition. One hypothesis was that higher-volume centers or those with specialized infrastructure (e.g. nutrition support teams) might implement evidence-based nutrition practices more rigorously. Our results provide partial support for this. We found that for esophagectomy and gastrectomy, a large proportion of responding hospitals were low-volume (performing fewer than 20 such surgeries per year), but practices did not differ significantly.

More clearly, the level of care and availability of specialized personnel had an influence on certain practices. Hospitals identified as higher-tier (e.g. university or tertiary referral centers) and those that reported having a dedicated nutrition support team were more likely to follow guidelines on specific points. Malnutrition screening rates were higher in centers with nutrition support teams, and high-volume centers also tended to allow oral intake earlier after colorectal surgery.

### Gaps between recommendations and practice

Our survey highlights a fundamental gap between guideline recommendations and actual practice in perioperative nutrition. Of the 29 nutrition-related items we surveyed, only two achieved what we defined as consensus implementation (adherence in >75% of hospitals)​. In this context, adherence reflects the extent to which clinical practice aligns with current guideline recommendations. This lack of consensus is not unique to Germany – it echoes a global challenge in surgical care [26]. Even well-established ERAS elements often see incomplete uptake. A 2023 international update on ERAS in gynaecologic oncology observed that “despite best efforts, many of the ERAS recommendations remain poorly adhered to and barriers to ERAS implementation persist” [27]. An Australian multicenter prospective study showed that the ESPEN guidelines were followed in only one-third of patients. Notably, adherence was less common in pancreatic surgery compared to gastric and esophageal surgery [25]. Our data in the domain of nutritional management reinforce this statement.

Understanding why guidelines are not followed is key to closing this gap. Prior studies have shed some light: UK clinicians cited organizational and timing issues (e.g. patients being referred too close to surgery to intervene on nutrition) and unclear responsibilities as barriers​ [2]. To bridge this implementation gap, several strategies emerge from our findings.

First, awareness has to be emphasized that nutritional status and body composition are prognostic for surgical and oncological as well as patient-reported outcomes.

Second, development of local protocols or care pathways can translate guidelines into precise steps at the hospital level. As we saw, centers with nutrition support teams have better adherence. Third, enhancing multidisciplinary involvement including tumour board is crucial. Information about nutritional status and body composition should be available and discussed regarding the indication for prehabilitation strategies.

### Limitations

Since participation in the survey was voluntary, there was a potential risk of selection bias. Voluntary participation can introduce selection bias because the sample may not accurately represent the target population. Individuals who chose to participate might have particular interest or experience related to the survey topic and might be more motivated to respond, skewing results. This self-selection could lead to over- or underestimation of associations or prevalence rates observed in the survey. Furthermore, given the large number of recipients, the limited number of responses suggests that mainly those with a particular interest in the topic chose to respond. As a limitation, it should be noted that responses may have been influenced by response bias, as responders may not wish to admit that their perioperative nutrition practices are outdated or inadequate. This implies that the true level of adherence may be even lower than our results suggest. Furthermore, responses were obtained from multiple staff members of the same institution in 43 cases. To avoid duplication, these responses were excluded, retaining only one dataset per clinic. Senior staff may portray their institution more positively than is typical in daily clinical practice, particularly in nutritional care. A random choice of one respondent per institution would have reduced this bias. Because the target group of the survey were surgeons, the questionnaire was validated only by five surgeons and one dietitian.

## Conclusion

While ERAS is standard of care including early oral nutrition, our national survey exposes substantial inconsistencies between recommended and actual practices in perioperative nutritional management in gastrointestinal cancer surgery across German hospitals. Malnutrition often goes unrecognized or untreated, and proven interventions like early feeding are underutilized in certain scenarios. The compliance with current international and national guidelines is low. These gaps have important implications: addressing them through concerted efforts could improve short and long-term outcomes of the surgical cancer patient on a broad scale. Multiprofessional teams, interdisciplinary rounds or clinical nutrition-focused meetings can ensure that nutrition assessment or timely initiation of feeding deserve higher priority, emphasizing the perioperative nutritional care path.

## Supplementary Information

Below is the link to the electronic supplementary material.

Supplementary Material 1 (DOCX. 140 KB)

## Data Availability

No datasets were generated or analysed during the current study.
