# Supplementary material for: Perioperative nutrition practices in gastrointestinal cancer surgery: A nationwide survey among German surgical departments
Source: Langenbecks Arch Surg. 2025 Nov 10;411(1):7. doi: 10.1007/s00423-025-03906-2 (PMC12602649; doi:10.1007/s00423-025-03906-2)
Supplement: Supplementary file 1 — Supplementary Material 1 (DOCX. 140 KB) [file 423_2025_3906_MOESM1_ESM.docx]

*Perioperative Nutrition Practices in Gastrointestinal Cancer Surgery: A Nationwide Survey among German Surgical Departments*

Rahel Maria Strobel^1^, Katharina Beyer^2^, Johannes Christian Lauscher^2^, Marc Martignoni^3^, Christoph Reißfelder^4^, Tim Vilz^5^, Arved Weimann^6^, Maria Wobith^7^

1. Department of General and Visceral Surgery, Charité – Universitätsmedizin Berlin, corporate member of Freie Universität Berlin und Humboldt-Universität zu Berlin, Campus Benjamin Franklin, Berlin, Germany
2. Department for General, Visceral and Transplantation Surgery, University Medicine Augsburg, Stenglinstraße 2, 86156 Augsburg, Germany
3. Department of Surgery, University Hospital rechts der Isar, Technical University of Munich (TUM), Ismaninger Str. 22, 81675 Munich, Germany
4. Department of Surgery, University Medical Center Mannheim, Medical Faculty Mannheim, Heidelberg University, Theodor-Kutzer-Ufer 1-3, 68167 Mannheim, Germany
5. Department of General, Visceral, Thoracic, and Vascular Surgery, University Hospital Bonn, Rheinische Friedrich-Wilhelms-University Bonn, Venusberg-Campus 1, 53127 Bonn, Germany
6. Department of General, Visceral, and Oncological Surgery, St. George Hospital, Leipzig, Germany
7. Department of Surgery, National University Hospital, Singapore

*Corresponding author:*

Dr. med. Rahel Maria Strobel

Department of General and Visceral Surgery

Charité – Universitätsmedizin Berlin

Campus Benjamin Franklin

Hindenburgdamm 30

12203 Berlin, Germany

Email: rahel.strobel@charite.de

Phone: 0049 (0)30-450-622727; Fax: 0049 (0)30-450-9522902

*Supplement*

Table 1 Characteristics of respondents

| **Variable** | **N = 263** |
| --- | --- |
| Age (median) | 47 years; 25% IQR: 38 years; 75% IQR: 55 years |
| What position do you work in hospital? |  |
| Senior physician | 99 (37.6%) |
| Head of department | 60 (22.8%) |
| Resident doctor | 32 (12.2%) |
| Head senior physician | 30 (11.4%) |
| Consultant doctor | 27 (10.3%) |
| Dietitian | 11 (4.2%) |
| Nutritionist | 4 (1.5%) |
| Which specialization do you have (more than one option possible)? |  |
| Visceral surgery | 200 (76.0%) |
| Specialized visceral surgery | 100 (38.0%) |
| Surgery | 85 (32.3%) |
| General surgery | 65 (24.7%) |
| Proctology | 52 (19.8%) |
| Nutritional medicine | 17 (6.5%) |
| In training | 14 (5.3%) |
| Which level of care of the hospital do you work in? |  |
| Regular care (300 to 499 beds) | 109 (41.4%) |
| Maximal care (700 to > 1000 beds) | 52 (19.8%) |
| Centralized care (500 to 699 beds) | 39 (14.8%) |
| University hospital | 30 (11.4%) |
| Standard care (200 to 299 beds) | 30 (11.4%) |
| Basic care (150 to 199 beds) | 3 (1.1%) |

Figure 1 Number of surgeries performed in the participating hospitals and the proportion certified by the German Cancer Society.

Figure 2 Numbers of oncological resections performed per hospital per year.

Table 2 Nutritional assessment

| **Variable** |  |
| --- | --- |
| **Methods used for nutritional assessment** | **N = 194** |
| Bioelectric impedance analysis | 28 (14.5%) |
| Subjective Global Assessment (SGA) | 19 (9.8%) |
| Mini Nutritional Assessment long-form | 16 (8.3%) |
| Imaging-based muscle mass and quality assessment (computed tomography, ultrasound or magnetic resonance imaging | 10 (5.2%) |
| Global Leadership in Malnutrition criteria (GLIM) | 7 (3.6%) |
| Patient-generated subjective global assessment (PG-SGA) | 2 (1%) |
| Alternative measures (BMI, laboratory results, serum albumin levels, muscle strength assessment) | 14 (7.3%) |
|  |  |
| **When do you perform calculation of caloric requirement?** | **N = 76** |
| Preoperatively | 31 (40.8%) |
| On the first postoperative day | 23 (30.3%) |
| Only in specific cases | 20 (26.3%) |
| In case of complications | 13 (17.1%) |
| Postoperatively before discharge | 11 (14.5%) |
|  |  |
| **When do you perform calculation of protein requirement?** | **N = 47** |
| Preoperatively | 20 (42.6%) |
| On the first postoperative day | 16 (34.0%) |
| In case of complications | 12 (25.5%) |
| Only in specific cases | 12 (25.5%) |
| Postoperatively before discharge | 8 (17.0%) |

Table 3 Decision making when oral feeding is not allowed on POD 1

| **Patients who are not allowed to eat on POD 1** |  |
| --- | --- |
| **Colorectal** | **N = 12 of 215 overall (5.6%)** |
| When are patients allowed to eat then? (more than one option possible) |  |
| On the second POD | 6 (50.0%) |
| On the third POD | 2 (16.7%) |
| Individually | 4 (33.3%) |
| **Pancreatic surgery** | **N = 44 of 113 overall (38.9%)** |
| When are patients allowed to eat then? (more than one option possible) |  |
| On the second POD | 10 (22.7%) |
| On the third POD | 4 (9.1%) |
| After the third POD | 10 (22.7%) |
| Based on surgeons’ discretion | 13 (29.5%) |
| Individually based on patients’ tolerance | 16 (36.4%) |
| After removal of gastric tube | 7 (15.9%) |
| **Gastric surgery** | **N = 82 of 170 (48.2%)** |
| When are patients allowed to eat then? (more than one option possible) |  |
| On the second POD | 21 (25.6%) |
| On the third POD | 24 (29.3%) |
| On the fourth POD | 6 (7.3%) |
| After the fourth POD | 15 (18.3%) |
| After checking anastomosis via endoscopy or contrast swallow | 27 (32.9%) |
| **Oesophageal surgery** | **N = 42 of 52 (80.8%)** |
| When are patients allowed to eat then? (more than one option possible) |  |
| On the second POD | 4 (9.4%) |
| On the third POD | 2 (4.8%) |
| After the third POD | 10 (23.8%) |
| Based on surgeons’ discretion | 18 (42.9%) |
| Individually based on patients’ tolerance | 11 (26.2%) |
| After removal of gastric tube | 4 (9.5%) |
| After checking anastomosis via endoscopy or contrast swallow | 21 (50%) |

Figure 3 Overview on management of postoperative parenteral feeding.
